# Zebrafish response to a robotic replica in three dimensions

**DOI:** 10.1098/rsos.160505

**Published:** 2016-10-19

**Authors:** Tommaso Ruberto, Violet Mwaffo, Sukhgewanpreet Singh, Daniele Neri, Maurizio Porfiri

**Affiliations:** Department of Mechanical and Aerospace Engineering, New York University Tandon School of Engineering, Brooklyn, NY 11201, USA

**Keywords:** binary choice test, information theory, robotics, zebrafish, three-dimensional tracking

## Abstract

As zebrafish emerge as a species of choice for the investigation of biological processes, a number of experimental protocols are being developed to study their social behaviour. While live stimuli may elicit varying response in focal subjects owing to idiosyncrasies, tiredness and circadian rhythms, video stimuli suffer from the absence of physical input and rely only on two-dimensional projections. Robotics has been recently proposed as an alternative approach to generate physical, customizable, effective and consistent stimuli for behavioural phenotyping. Here, we contribute to this field of investigation through a novel four-degree-of-freedom robotics-based platform to manoeuvre a biologically inspired three-dimensionally printed replica. The platform enables three-dimensional motions as well as body oscillations to mimic zebrafish locomotion. In a series of experiments, we demonstrate the differential role of the visual stimuli associated with the biologically inspired replica and its three-dimensional motion. Three-dimensional tracking and information-theoretic tools are complemented to quantify the interaction between zebrafish and the robotic stimulus. Live subjects displayed a robust attraction towards the moving replica, and such attraction was lost when controlling for its visual appearance or motion. This effort is expected to aid zebrafish behavioural phenotyping, by offering a novel approach to generate physical stimuli moving in three dimensions.

## 1. Introduction

In recent years, zebrafish (*Danio rerio*) has been extensively used in laboratory experiments for the investigation of a broad spectrum of functional and dysfunctional processes, such as neurological disorders [1], vertebrate embryonic development [2,3], preclinical toxicological assessment [4,5] and pharmacological assays [6]. The increasing use of zebrafish in animal experiments is driven by several factors, including their fully sequenced genome [7–9], rapid development from larval to adult stages [10,11] and reduced maintenance cost [12]. A deeper understanding of social behaviour in zebrafish may help the design of experimental models and protocols, aiding the comprehension of the fundamental mechanisms behind the onset of human neurobehavioural disorders such as anxiety, addiction to substances of abuse, autism spectrum disorders and schizophrenia [13]. Although significant progress has been made in phenotyping their behaviour [14], the neurobiological determinants of zebrafish sociality are not yet fully apprehended.

A battery of experimental tests has been devised to examine the social behaviour of this freshwater species, often borrowing from well-established paradigms used for rodents [15–18]. A popular paradigm consists of studying the spatial preference of zebrafish in binary choice tests, using live [17,19,20] or video stimuli [21–23]. In these experiments, fish behaviour is often scored in terms of time spent in the proximity of the stimuli [24], the average distance between fish and stimuli [25] or the number of entries in specific tank sections [26]. These quantities are typically studied by recording zebrafish swimming patterns in two dimensions and analysing the acquired videos manually or through automated software [8].

The binary choice test has helped us to significantly improve our understanding of zebrafish social behaviour, including attraction towards conspecifics or heterospecifics [25,27] and the role of shoal size [28], sex [29], colour pattern [24] and interindividual distance [30]. However, both live and video stimuli may suffer from methodological confounds, whereby (i) live stimuli may result in large intrinsic variability among experimental trials owing to idiosyncrasies with focal subjects, tiredness and circadian rhythms, and (ii) video stimuli only offer two-dimensional projections of images and suffer from the absence of a physical input for fish to interact with [31].

To overcome these limitations, the use of robotic stimuli has been proposed as an alternative approach [32]. In these experiments, robotic stimuli are typically developed, using three-dimensional printing techniques to replicate the morphology of zebrafish and mimic their swimming patterns. When compared with video stimuli, they allow for a higher degree of control and a more realistic interaction [33], which are accompanied by a superior level of consistency with respect to live stimuli [33,34]. These key methodological advantages have also been confirmed in the study of other freshwater species and experimental paradigms, fuelling intense research on guppies [35,36], golden shiners [37–39], mosquitofish [40], sticklebacks [41], killifish [42] and giant danios [43].

Although these studies have significantly contributed to demonstrating the potential of robotics in zebrafish behavioural phenotyping in choice tests, they either rely on stimuli anchored at a fixed location of the tank while beating their tail [27,33,44–47] or on replicas which are dragged along periodic linear or figure-eight trajectories in two dimensions using external manipulators [33,48,49]. Here, we seek to further advance the use of robotic stimuli in zebrafish experimentation through a novel four-degree-of-freedom platform, which affords three-dimensional motions as well as body oscillations. The platform can be pre-programmed to actuate a three-dimensionally printed replica that closely mimics zebrafish morphology along a three-dimensional trajectory, summarizing several elements of the behavioural repertoire of zebrafish observed in experimental studies on isolated fish in tanks, such as thrashing against walls, erratic movements and diving, and freezing bouts [14].

The platform is integrated with a custom-made image processing software developed by our group to enable three-dimensional trajectory reconstruction [33], which is central in refined behavioural phenotyping [8,50,51]. Not only are three-dimensional measurements expected to afford the accurate measurement of a number of key behavioural observables, but they should also unveil components of the ethogram that would not be discovered from two-dimensional data [50].

To demonstrate the use of the proposed platform and investigate the interaction of zebrafish with biologically inspired three-dimensionally actuated robotic replicas, we conducted a series of experiments in a binary choice test. Similar to well-established protocols that are based on the use of live stimuli, we physically separated the focal subjects from the robotic replicas using transparent panels [52]. We considered five experimental conditions to isolate the effects of the motion of the replica and its visual appearance. In these conditions, live subjects interacted with a three-dimensionally moving replica; a two-dimensionally moving replica; a non-moving, static replica; an optically transparent three-dimensionally moving model of the replica and a non-moving rod without any stimulus attached.

We examined the behavioural response of zebrafish in terms of their time budgeting in the tank (along the length and depth of the tank), shoaling tendency with respect to the stimulus and activity (average speed and acceleration). To refine and enhance the analysis of the interaction between the fish and the replica, we used the information-theoretic notion of transfer entropy [53]. Transfer entropy has been shown to offer a valid complement to traditional indicators of fish spatio-temporal preference, revealing the influence of robotic stimuli on fish behaviour [49,54,55].

## 2. Material and methods

### 2.1. Animals and housing

A total of 50 zebrafish (*D. rerio*) of wild-type variety, approximately 3 cm in total body length, were used in this study. Fish were purchased from an online aquarium vendor (LiveAquaria.com, Rhinelander, WI) and housed in 37.8 l (10 gallon) tanks, with a maximum of 20 fish per tank. Prior to the experiments, they were acclimatized for a period of 12–15 days in housing tanks where temperature and acidity were maintained at 26°C and pH 7.2, respectively. Fish were kept under a 12 L : 12 D photoperiod [56] and fed with commercial flake food (Hagen Corp. Nutrafin max) between 18.00 and 19.00 every day.

### 2.2. Robotic platform and replica

**Figure 1.**
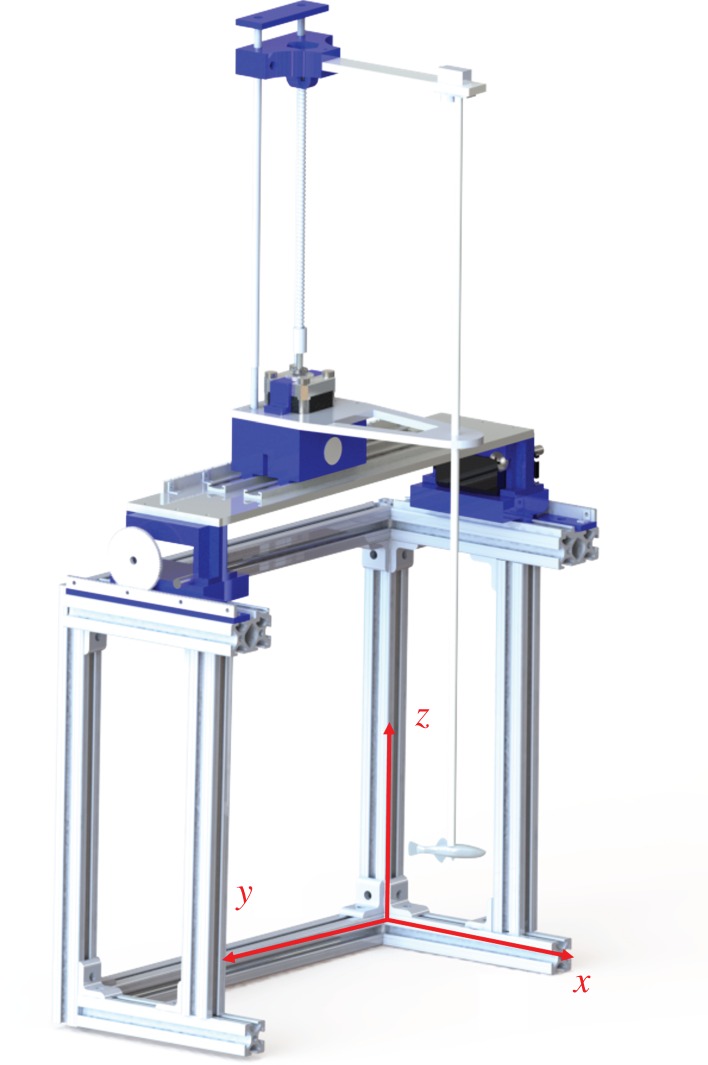
Robotic platform used to manoeuvre the zebrafish replica in three dimensions, while oscillating about the vertical axis.

The robotic platform (figure 1) was designed in SolidWorks (Dassault Systèmes SolidWorks Corp., Waltham, MA) and is composed of aluminium T-slot bars (McMaster Carr, Elmhurst, IL). The platform was operated with an Arduino microcontroller (Arduino Uno, Arduino, Italy) connected to a computer using an ethernet shield, communicating using the user datagram protocol sent from a program written in Matlab (MathWorks, Natick, MA) running on the computer.

**Figure 2.**
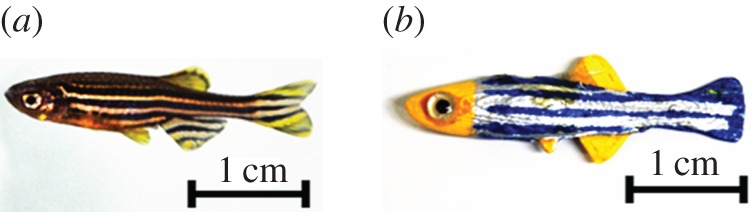
Live swimming zebrafish (*a*) and fish replica painted to resemble a zebrafish (*b*).

The robotic fish replica was designed to mimic zebrafish morphology. Specifically, it has dorsal, ventral and caudal fins, and it was coloured using non-toxic colours (Krylon, Krylon Products Group, Cleveland, OH) with stripes resembling the body pattern of a live zebrafish (figure 2). Glass eyes (Van Dyke Supply Co., Granite Quarry, NC) were attached to the replica to offer a realistic eye appearance, which has been found to be critical in other studies [36]. The replica was fabricated using acrylonitrile butadiene styrene thermoplastic in a Dimension Elite three-dimensional printer (Stratasys Ltd., Eden Prairie, MN).

The replica was attached to an acrylic rod on the robotic platform and programmed to move in three dimensions, denoted *x, y* and *z* (see videos in the electronic supplementary material). The motion of the replica along the *x*-axis was driven by two servomotors (Futaba Corporation of America, Schaumburg, IL) attached to a system of rack-and-pinions. The *y*-axis motion was actuated through a DC motor (Robotzone, LLC, Winfield, KS), which was also connected to a system of rack-and-pinions. The motion on the *z*-axis was generated using a threaded rod, which was actuated by a stepper-motor (Adafruit, New York City, NY). The orientation of the rod, and subsequently, the orientation of the replica, was controlled by a small Hitec servomotor (Hitec RCD USA, Inc.) attached to the cantilever travelling on the threaded rod.

### 2.3. Apparatus

**Figure 3.**
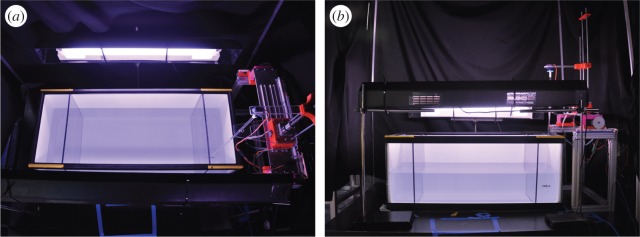
Top (*a*) and front (*b*) views of the experimental set-up. The stimulus is placed in one of the two small side compartments, keeping the other one empty, whereas the focal fish is released in the middle compartment.

The experimental set-up (figure 3) consisted of a water tank of dimensions 74 × 30 × 30 cm (length, width and depth) divided into three compartments using Plexiglas panels, with the two lateral partitions of 10 × 30 × 30 cm (length, width and depth). The water depth was kept at 15 cm. White contact paper was applied to the walls of the tank, with the exception of the front side which was used to video record fish motion along the water column.

The water tank was illuminated using two 25 W fluorescent tubes (All-Glass Aquarium, UK) mounted along the two longitudinal sides. The lights were positioned 30 cm above the surface of the water. Experiments were video recorded using two Logitech web cameras (Logitech, Newark, CA) placed above the tank at a distance of 55 cm from the water surface as well as in front of the tank at a distance of 50 cm, to enable three-dimensional reconstruction of zebrafish motion. Black curtains surrounded the experimental set-up on all sides, in order to isolate the experimental set-up from any external source of light.

The two cameras were activated simultaneously, using a Linux shell script, and calibrated with the Matlab calibration toolbox (R2011a, MathWorks). Videos were post-processed offline by first converting them into image frames, and then using a multi-target tracking algorithm, implemented in Matlab R2014b (MathWorks), to estimate two-dimensional trajectories of fish and stimuli.

**Figure 4.**
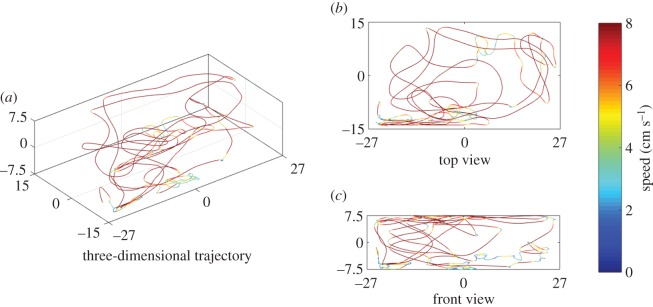
Sample fish trajectory reconstructed over 120 s in three dimensions (*a*), top view (*b*) and front view (*c*). The colouring in (*b*,*c*) indicates fish instantaneous speed, as shown by the colour bar.

The tracking software estimated the three-dimensional position of the fish and the stimuli after applying appropriate filtering procedures to two-dimensional observations from each view [55]. Briefly, a simple background subtraction was performed on each image frame to identify the centroid of the segmented target blobs corresponding to the fish and the stimuli [55]. Trajectory data from each view were further smoothened, using a simple moving average filter with a window size of 18 frames to suppress noise from tracking, selected on the basis of comparison with previous work [33]. Assuming that the two-dimensional views were orthogonal, the trajectories for the top and the front views (figure 4*b,c*) were then concatenated to estimate the three-dimensional trajectory of the fish and the stimulus (figure 4*a*). To ease the tracking of the stimulus, especially the transparent model, the process of synchronization was partially automated. Specifically, we conducted a separate trial in which we filmed only the moving replica in three dimensions without live subjects and Plexiglas panels, and we used this trial to assist in the synchronization of the two views.

### 2.4. Experimental procedure

Experiments were performed between August and September 2015 and June and July 2016. The focal fish placed in the middle compartment was presented with the replica operated by the robotic platform on the one side and an empty compartment on the other (figure 3). After the focal fish was released in the tank with a hand net, the cameras started recording for 20 min at 30 frames rate per second, comprising 10 min of habituation and 10 min of experimental observation.

**Figure 5.**
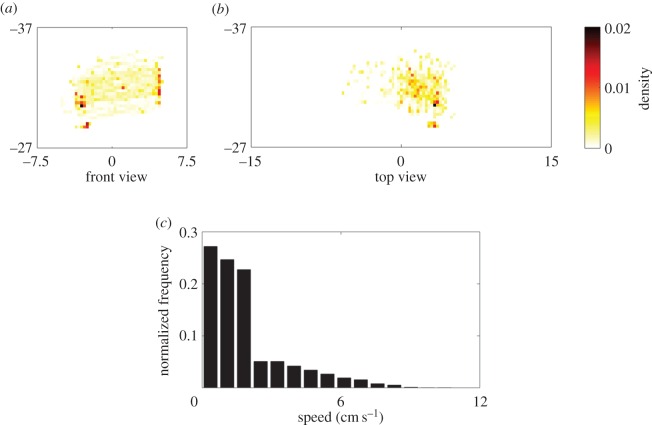
Salient characteristics of the replica motion, including (*a*) position density in the front view, (*b*) position density in the top view and (*c*) distribution of the speed. The entire 10 min duration is used for the computations; density is computed using a two-dimensional bin size of 0.37 times 0.37 cm; and the speed distribution uses a bin size of 0.72 cm s^−1^.

Five experimental conditions were executed. In condition ‘replica + motion’, RM, a replica was attached to the rod, which was actuated by the robotic platform to replicate the motion of a live subject. The trajectory of the rod was programmed based on the trajectory of a live zebrafish that was observed swimming in the lateral compartment of the tank for a period of 10 min after 10 min of habituation. The 10 min of observation was tracked in three dimensions and then downsampled. Specifically, the positions of the live fish along the *x*, *y* and *z* axes were sampled every 2.5 s from the original trajectory. The resulting coordinates were assembled in a single trajectory, which was used to actuate the rod in each and every trial (figure 5).

The motion of the replica included instances of freezing, thrashing against the Plexiglas wall subdividing the experimental tank, erratic movement and diving. The average speed was 1.69 cm s^−1^, and the average position along the water column was 7.56 cm below the water surface, almost in the middle of the water column. Its heading was programmed to either face the centre of the tank or face away from it, while oscillating at 2 Hz with an angular peak-to-peak amplitude of 10° to mimic zebrafish body movement [54]. Specifically, the average heading of the replica with respect to the *x*-axis was 45° or 135°, depending on the direction of motion along the *y*-axis.

Condition ‘replica + motion in two dimensions’, RM 2D, controlled for the three-dimensional motion of the rod, whereas conditions ‘motion’, M, and ‘replica’, R, controlled for either the visual appearance of the replica or the imposed motion to the rod. Specifically, in condition RM 2D, a replica was attached to the rod, which was actuated by the platform along a two-dimensional trajectory generated by blocking the motion of the RM trajectories along the *z*-axis at 7.50 cm below the water surface. In condition M, a transparent model of the replica was hand carved from acrylic plastic and connected to the rod that was manoeuvred as in condition RM. In condition R, a replica was used facing the centre of the tank, along the *x*-axis, and the rod was held fixed with the replica positioned in the middle of the tank at 7.50 cm below the water surface. Finally, we performed one control condition, Control, where neither a replica nor a transparent model were attached to the rod. Therein, the rod was not moving and held just below (1 mm) the water surface.

In conditions RM, RM 2D and M, the rod started moving within 1 s after the recording had begun. Each condition was conducted using 10 experimentally naive subjects, resulting in 50 trials. At the end of the recording time, the fish was removed from the experimental tank with a hand net and placed into a separate tank. In conditions R and Control, the rod was positioned at the centre of the lateral compartment, with the replica facing the centre of the tank when present. For each condition, five fish were tested in the morning and five in the afternoon for a total of 10 fish per condition, between 10.00–13.30 and 14.00–18.30, respectively. The position of the platform (left or right compartments) was balanced across trials in each condition.

### 2.5. Data analysis

Fish activity was scored in terms of average speed and acceleration, computed from the estimated three-dimensional motion. Velocity was computed using first-order numerical differentiation of the fish position time series, filtered using a simple moving average of 18 frames window size. Acceleration was then evaluated from velocity data, again using a first-order numerical differentiation.

To analyse the spatial preference of the focal fish for the stimuli, the central compartment was virtually divided along its length into three equal sections of 18 cm. Time spent in the section near the stimulus (*T*_N_), in the middle section, in the farthest section (*T*_F_) were computed from the reconstructed three-dimensional motion. Fish preference for the stimulus was quantified through the preference index (PI) [16,33,46], defined as *T*_N_/(*T*_N_ *+* *T*_F_).

A similar approach was followed to examine time budgeting along the water column, by partitioning the water column in three equal levels of 5 cm. We measured the time spent in the bottom (*T*_B_), middle and top (*T*_T_) level. We aggregated the time spent in the top and bottom in a PI for the bottom level as defined as *T*_B_/(*T*_B_ *+* *T*_T_).

Fish shoaling tendency was computed from the three-dimensional trajectories as the time spent by fish within four body lengths of the stimulus (12 cm), similar to [57]. (The presence of the partition constrains the minimum distance between the stimulus and the focal subject.) In the control condition, where no stimulus was used, the shoaling tendency was measured with respect to the trajectories of the replicas in condition RM. For each of the control subjects, we computed the time that a fish would spend within four body lengths of the moving replica.

The interaction between the fish and the moving stimuli (conditions RM, RM 2D and M) was further quantified using transfer entropy, an information-theoretic quantity that can be used to estimate causal relationships between two time series [53]. Specifically, given two discrete stochastic processes, *X* and *Y*, modelled as a Markov chain of order one, the transfer entropy from *Y* to *X* is given by

2.1 $$T_{Y\to X}=\underset{x_{t+1},x_{t},y_{t}}{\sum }p(x_{t+1},x_{t},y_{t})\;\log_{2}\frac{p(x_{t+1}|x_{t},y_{t})}{p(x_{t+1}|x_{t})},$$

where *x_t_* and *x_t + _*_1_ are the outcomes of *X* at time step *t*, and *t* + 1, *y_t_* is the outcome of *Y* at *t*, and Σ denotes the summation over all possible realizations of *x_t_*, *x_t + _*_1_ and *y_t_*; *p*(*x_t + _*_1_|*x_t_*, *y_t_*) is the conditional probability density of *X* at *t* + 1, given its previous value and that of the process *Y*. This quantity should be taken as direct measurement of the reduction in uncertainty of the stochastic process *X* given the knowledge of *Y*. As a result, by contrasting *T_Y _*_→ *X*_ and *T_X _*_→ *Y*_, we may assess the direction of the dominant information transfer between the processes or, equivalently, dissect cause-and-effect relationships.

Transfer entropy was computed using Process_Network_v. 1.4 software [58] for RM, RM 2D and M conditions. Trajectory data were initially filtered using a wavelet filter implemented in Matlab 2014b and then segmented such that only portions of the trajectories in the virtual section near the stimulus were retained. Following our previous work [48], transfer entropy was evaluated only for segments containing at least 175 data points, using the position of the fish and the stimulus along the width of the tank as the input signals. The computation was executed using 10 bins, corresponding to a bin size approximately equal to one body length.

To ensure the validity of the measured information transfer, we computed transfer entropy between live subjects in the control condition and the replica in condition RM. Given that control subjects were not exposed to the motion of the stimulus, we sought to verify the absence of information transfer between fish and replica.

Outliers' values were identified using the interquartile range [59] applied to the time spent in each lateral section. Seven fish, one in condition RM, one in condition RM 2D, three in condition R and two in condition M, were consistently discarded from the analysis. For each condition, a one-sample *t*-test with unknown variance and reference mean equal to 50% was performed to evaluate the spatial preference of the fish for the stimulus and for the bottom level. Similarly, one-way ANOVA was used to compare the shoaling tendency in each condition with the baseline attraction for the sides of the tank.

One-way ANOVA was also used to compare observables (PI for the stimulus and the bottom level of the tank, time spent in the middle section along the length of the tank, time spent in the middle of the water column, average speed, average acceleration and shoaling time) with the experimental condition as the independent variable. In case of significance, a Tukey's honestly significant difference criterion post hoc test was used for pairwise comparisons. The direction of information transfer was assessed, using repeated measures ANOVA for each of the three experimental conditions with a moving stimulus (RM, RM 2D and M), with data segments as within group. One-way ANOVA was used to compare the transfer entropy from stimuli to fish between RM, RM 2D and M conditions. Finally, one-way ANOVA was used to compare transfer entropies between live subjects in the control condition and the replica in conditions RM, RM 2D and M. All the analyses were conducted with a significance level set at 0.05, unless otherwise specified.

## 3. Results

### 3.1. Spatio-temporal preference

**Figure 6.**
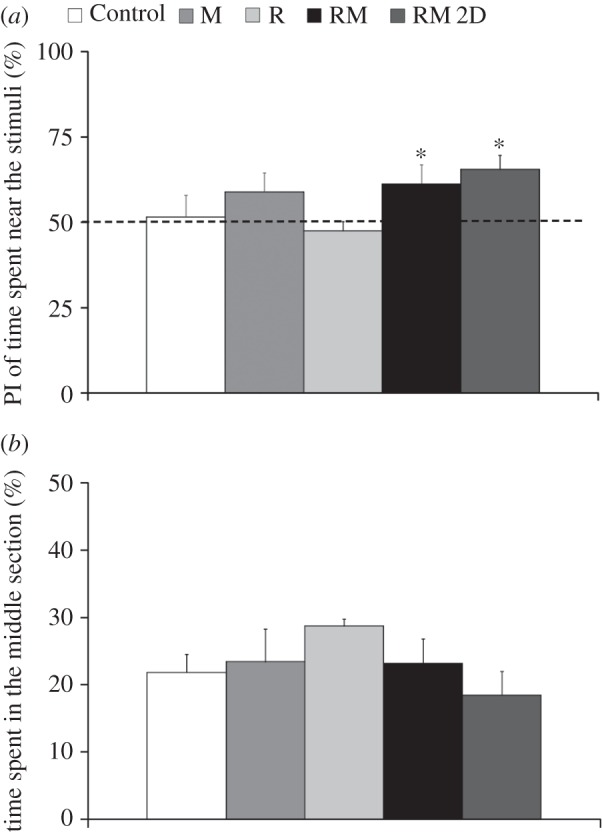
Average preference index (PI) for the time spent in the stimulus section (*a*), corresponding to one-third of the experimental tank, and average time spent in the middle section of the tank (*b*). In condition RM, fish are presented with the moving replica; in condition RM 2D, fish are presented with the replica moving in two dimensions; in condition R, the replica is held fixed; and in condition M a transparent model of the replica is attached to the moving rod. Asterisk indicates *p* < 0.05 in one-sample *t*-test comparison with respect to chance, indicated as a dashed line. Error bars represent ± standard errors.

Fish were attracted (figure 6*a*) by the replica moving in three dimensions (*t*_8_ = 1.98, *p* = 0.0413) and in two dimensions (*t*_7_ = 3.84, *p* = 0.0031). No preference was found for the stimulus in conditions R (*t*_6_ = 0.92, *p* = 0.1954), M (*t*_8_ = 1.31, *p* = 0.1128) and Control (*t*_9_ = 0.24, *p* = 0.8163). One-way ANOVA comparisons did not indicate a difference between conditions with respect to their preference for the stimulus section (*F*_4,38 _= 1.54, *p *= 0.2090). Similarly, no difference in time spent by the fish in the central section of the tank (figure 6*b*) was found across experimental conditions (*F*_4,38_ = 0.97, *p *= 0.4372).

**Figure 7.**
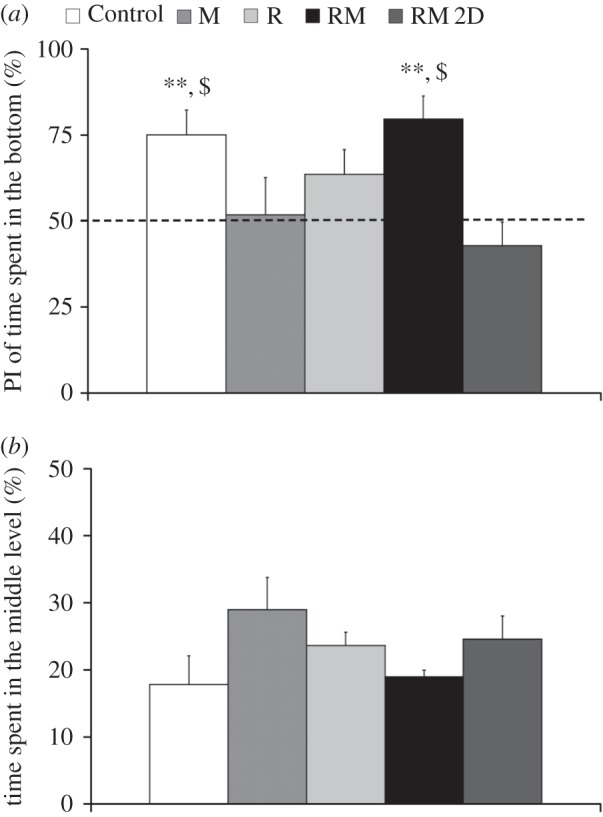
Average preference index (PI) for the time spent in the bottom level of the tank (*a*), corresponding to one-third of the water column, and average time spent in the middle level of the water column (*b*). In condition RM, fish are presented with the moving replica; in condition RM 2D, fish are presented with the replica moving in two dimensions; in condition R, the replica is held fixed; and in condition M a transparent model of the replica is attached to the moving rod. Asterisks indicate *p* < 0.01 in one-sample *t*-test comparison with respect to chance, indicated as a dashed line. Dollar symbol indicates significance in post hoc comparisons (*p* < 0.05) with condition RM 2D. Error bars represent ± standard errors.

Along the water column, focal fish displayed a robust preference (figure 7*a*) for the bottom level of the tank in conditions RM (*t*_8_ = 4.46, *p* = 0.0010) and Control (*t*_9_ = 3.51, *p* = 0.0033). No preference for the bottom of the tank was found in conditions RM 2D (*t*_7_ = 1.05, *p* = 0.1636), R (*t*_6 _= 1.88, *p* = 0.0543) and M (*t*_8_ = 0.16, *p* = 0.4369). One-way ANOVA comparisons confirmed a significant difference between experimental conditions with respect to time budgeting along the water column (*F*_4,38_ = 3.79, *p* = 0.0109). Pairwise post hoc comparisons indicate a difference between the preference for the bottom of the tank in conditions RM and Control with respect to condition RM 2D (*p* < 0.05). One-way ANOVA comparisons did not indicate a difference in the time spent by the fish in the middle of the tank (figure 7*b*) across experimental conditions (*F*_4,38_ = 1.37, *p* = 0.2619).

### 3.2. Activity

**Figure 8.**
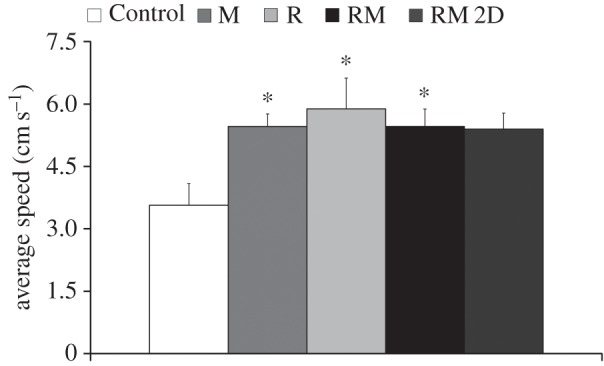
Average speed for the five experimental conditions. In condition RM, fish are presented with the moving replica; in condition RM 2D, fish are presented with the replica moving in two dimensions; in condition R, the replica is held fixed; and in condition M a transparent model of the replica is attached to the moving rod. Asterisk indicates significance in post hoc comparisons (*p* < 0.05) with the Control condition. Error bars represent ± standard errors.

**Figure 9.**
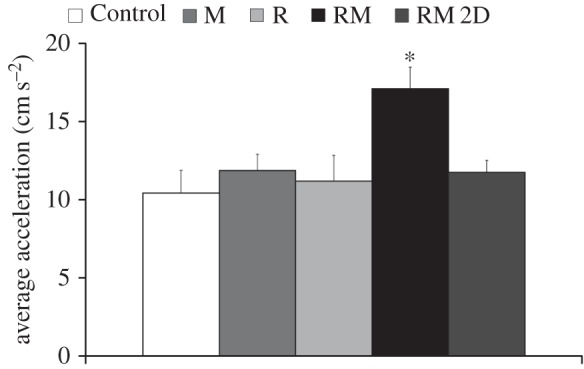
Average acceleration for the five experimental conditions. In condition RM, fish are presented with the moving replica; in condition RM 2D, fish are presented with the replica moving in two dimensions; in condition R, the replica is held fixed; and in condition M a transparent model of the replica is attached to the moving rod. Asterisk indicates significance in post hoc comparisons (*p* < 0.05) with the Control condition. Error bars represent ± standard errors.

Fish average speed (figure 8) was found to vary across conditions (*F*_4,38_ = 3.89, *p* = 0.0095), with fish in condition RM, R and M swimming faster than control subjects (*p* < 0.05 in post hoc comparison). Similarly, fish average acceleration (figure 9) significantly varied across conditions (*F*_4,38_ = 4.37, *p* = 0.0052), with fish in condition RM displaying a higher acceleration than subjects from other conditions (*p* < 0.05 in post hoc comparison).

### 3.3. Shoaling tendency

**Figure 10.**
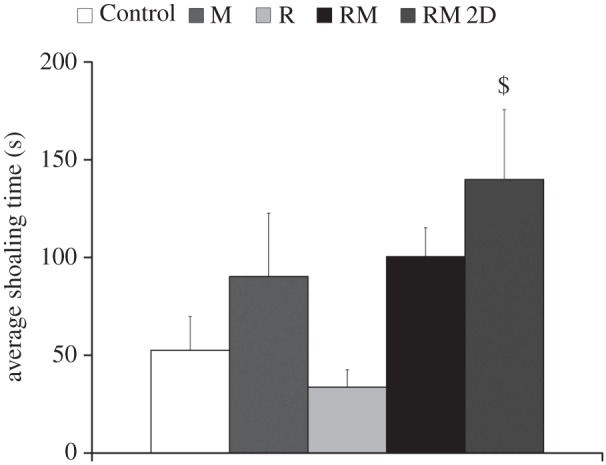
Average shoaling time for the five experimental conditions. In condition RM, fish are presented with the moving replica; in condition RM 2D, fish are presented with the replica moving in two dimensions; in condition R, the replica is held fixed; and in condition M a transparent model of the replica is attached to the moving rod; in Control condition, the motion of the replica in condition RM was used as a virtual stimulus. Dollar symbol indicates significance in post hoc comparisons (*p* < 0.05) with condition R. Error bars represent ± standard errors.

Variations in fish shoaling tendency (figure 10) were found across experimental conditions (*F*_4,38_ = 2.73, *p* = 0.0429), although none of the experimental conditions were different from Control in post hoc comparisons. Only significant difference was noted between conditions RM 2D and R (*p* < 0.05).

### 3.4. Information transfer

**Figure 11.**
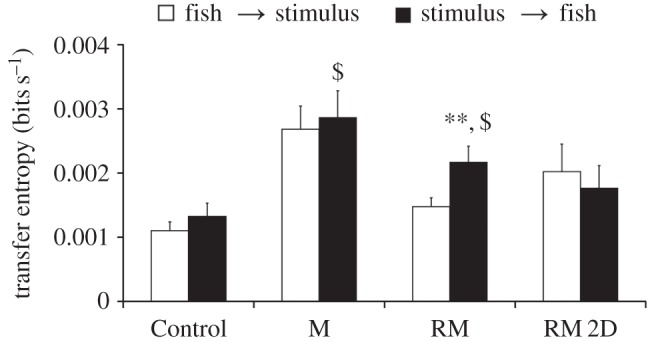
Information transfer from focal fish to stimuli and from stimuli to focal fish for the three experimental conditions with moving stimuli (RM, moving replica; RM 2D, replica moving in two dimensions; and M, moving transparent model of replica) and for the Control condition using the motion of the replica in condition RM as a virtual stimulus. Transfer entropy data for each segment are normalized with respect to the corresponding segment data length. Asterisks indicate significant difference in information transfer direction at *p* < 0.01, whereas dollar symbol indicates a significant difference with respect to the Control condition at *p* < 0.05. Error bars represent ± standard errors.

Information transfer (figure 11) was found to be significantly higher from replica to fish than from fish to replica in condition RM (*F*_1,205_ = 7.64, *p* = 0.0063) and not significantly different between directions in conditions M (*F*_1,93_ = 0.05, *p* = 0.8231) and RM 2D (*F*_1,81_ = 0.62, *p* = 0.4352). No difference in information transfer was also found in condition Control (*F*_1,201_ = 0.41, *p* = 0.5233), adapting the motion of the replica in condition RM as a virtual stimulus. A segment effect was found for transfer entropy in conditions RM 2D (*F*_7,81_ = 4.64, *p* = 0.0003) and RM (*F*_8,205_ = 2.07, *p* = 0.0403), whereas such an effect was not found in conditions M (*F*_8,93_ = 0.97, *p* = 0.4637) and Control (*F*_9,201_ = 0.98, *p* = 0.4541). No interaction effect between the direction of the information transfer and segments was found for transfer entropy in all conditions: RM (*F*_8,205_ = 0.75, *p* = 0.6448), RM 2D (*F*_7,81_ = 0.49, *p* = 0.8399), M (*F*_8,93_ = 0.48, *p* = 0.8684) and Control (*F*_8,201_ = 0.64, *p* = 0.7651).^1^

Pairwise comparisons on information transfer with respect to control subjects demonstrate that the moving replica in condition RM and the transparent model in condition M transferred more information than the virtual stimulus in condition Control (*F*_1,203_ = 14.42, *p* = 0.0002) and (*F*_1,147_ = 24.68, *p* < 0.0001), respectively, whereas no difference was found between RM 2D and the virtual stimulus in condition Control (*F*_1,141_ = 1.71, *p* = 0.1935).

## 4. Discussion

Here, we studied the behavioural response of zebrafish to a biologically inspired three-dimensionally printed replica, which was fabricated and painted to mimic zebrafish morphology. The replica was manoeuvred by a novel four-degree-of-freedom robotic platform, which was designed to move the replica along sampled three-dimensional trajectories of live subjects and, independently, control its body oscillation. A custom-made three-dimensional tracking software was used to examine the interaction between zebrafish and the stimuli. To isolate the effect of the motion from the visual appearance, five experimental conditions were performed, in which fish preference was tested against a three-dimensionally moving replica, a two-dimensionally moving replica, a fixed replica, a three-dimensionally moving transparent model of the replica and an immobile rod without a replica or a transparent model attached.

Our findings demonstrate that fish were attracted towards the three-dimensionally moving replica, and this attraction was lost when either its visual appearance or motion was controlled. Specifically, live fish did not display a preference for either the replica held fixed in the tank or the moving transparent model. The attraction towards the moving replica, quantified in terms of the time spent in its vicinity, was also accompanied by an increase in activity, measured through the average speed and acceleration, and a preference for the bottom of the tank. The interaction between the stimulus and the focal subjects was further evaluated through the information-theoretic construct of transfer entropy, which can be used to estimate causal relationships between coupled dynamical systems. Our results confirm that the three-dimensional motion of the replica influenced fish behaviour, and such an influence is abolished when the visual appearance or the motion along the water column are controlled.

Zebrafish rely primarily on visual cues in their social interactions with conspecifics and heterospecifics [14]. Stripe pattern, pigmentation and shape have been found to be determinant factors in the attraction of zebrafish for pigment pattern mutants and closely related species. Experiments using live, video and robotic stimuli have demonstrated a robust preference for fish with a similar morphology and may be repelled by subjects with elongated or compressed shapes and different stripe orientation or pigment [24,25,44]. The use of robotic stimuli has also helped clarifying the critical role of the stimulus motion on zebrafish behaviour, whereby fish presented with the choice between a static robot and one that was beating its tail preferred to spend time with the latter [45]. By controlling for visual stimulation in an experiment conducted in the dark, the same tail beating robot elicited avoidance response through auditory cues associated with the mechanical actuation. This offers indirect evidence for the central role of the visual perception of the stimulus motion on zebrafish behaviour, albeit potential confounds associated with multisensory stimulation.

In accordance with our expectations, we have found that removing visual cues associated with the presence of the biologically inspired replica suppresses the attraction towards the moving stimulus, measured through the PI for the stimulus partition. Transfer entropy also failed to identify an influence of the motion of the transparent model of the replica on the live subject. However, the transparent model was probably perceived by the subjects, as evidenced from the increase in the information transfer with respect to control subjects. The physical motion of the transparent model should be associated with visual and acoustic cues, beyond those offered by computer-animated images. It is possible to hypothesize visual signalling from the movement of the water generated by the translation of the model in the lateral compartment, along with acoustic signalling from the repeated collisions of the model with the walls during simulated thrashing. As expected, enriching the stimulus through a biologically inspired replica results in a net information transfer between the stimulus and the subject. This offers direct evidence for the role of visual cues in experiments using robotic stimuli.

In line with our predictions, we also confirmed the central role of the stimulus motion on zebrafish behaviour. Fish did not display attraction towards the static replica, whose only observed effect was to eliminate the preference for the bottom of the tank with respect to control subjects. This evidence could be related to the different vertical positioning of the cantilever along the threaded rod. Specifically, the lower distance of the cantilever in the condition with the static replica may reduce its visibility to live fish with respect to control subjects. The cantilever is likely to be seen by control subjects everywhere in the tank, owing to zebrafish enhanced visual acuity along oblique directions towards the water surface [60]. The cantilever could be associated by the focal subjects with an aerial predator, thereby increasing their tendency to swim in the bottom of the tank. Such a tendency could be further amplified by the view of the camera and part of its tripod from the open frontal side of the tank.

The response of zebrafish to a two-dimensionally moving replica allows for shedding light on the specific role of three-dimensional motion. The two-dimensionally moving replica was manoeuvred along a two-dimensional projection of the original three-dimensional trajectory at the middle of the water column. Although the two-dimensionally moving replica elicited a robust attraction towards live subjects, it did not modulate their activity and did not influence their motion. The increased activity displayed towards the three-dimensionally moving replica may be partially associated with the diving and the rising movements of the replica along the water column, which could be potentially amplified by the changing visibility of the cantilever travelling on the threaded rod. The diving and rising movements are also likely to play a role on the information transfer between the replica and the focal fish. The two-dimensionally moving replica will display a less dynamic stimulus, in which diving and rising movements are replaced by slower motions in the plane. Such motions could even result in freezing instances, especially close to the transparent partitions. Experiments in [49] seem to confirm the importance of fast changes in the stimulus trajectory, where a replica moving along a figure-eight trajectory was shown to influence zebrafish behaviour, without eliciting a robust preference.

Both the two- and three-dimensionally moving replicas indicate the presence of undesired time effects, related to the variability of the RM during a trial. Specifically, during a trial, a fish may be presented with different cues; for example, the replica may be thrashing against the partition or swimming on the opposite side in two separate time segments. Creating an array of trajectories for the replica and randomly selecting from them for each trial could potentially mitigate such time effects, although it may cause undesired variations in the treatment of each subject.

Not only does the platform constitute a technical advancement for studying zebrafish behaviour, but also it offers the possibility to robustly validate the information-theoretic construct of transfer entropy in the study of animal behaviour. Different from previous studies [48,49,54] featuring periodic motions with low information content, the proposed platform allows for generating highly irregular, aperiodic, information-rich motions that attempt at summarizing the complexity of zebrafish swimming. The information transfer between the three-dimensionally moving replica and fish offers strong evidence for the existence of a causal relationship at the basis of the fish–replica interaction, which could only be conjectured using our previous experiments, using periodic motions. Our results add to the growing literature on the use of transfer entropy to study social behaviour using experimental observations in laboratory [61] or field settings [62], as well as synthetic datasets generated using computer simulations [63–65].

Another contributing factor to the effect of the three-dimensionally moving replica could be the improved development of the replica. Compared with previous experiments, the stripe pattern has been more carefully painted and glass eyes were used instead of painted eyes. Based on recent observations on guppies [36], it is possible to hypothesize an enhanced attraction owing to the glass eyes. However, our experimental set-up differs from [36], where fish were allowed to freely interact with the robotic stimulus in an open tank rather than through a Plexiglas partition. This choice was motivated by three factors. First, we sought to prevent any physical damage to the focal fish related to the motion of the transparent rod, which operates in open-loop, independently of the fish position. Second, we sought to mirror the current practice in preference tests using live stimuli, in which physical partitions are also used to separate animals [52]. Third, our previous experiments on the interactions between live fish and robots anchored at precise locations in the tank (equivalent to the current platform without the *xyz* motion) suggest that the presence of the partition does not have a primary role on the interaction, which is instead highly influenced by the oscillation of the robot body [44,45]. These studies, however, used a stimulus with different features. Future studies will seek to elucidate the specific role of the eyes, stripe pattern and body oscillation of the present replica, through controlled experiments, targeting also the role of visual acuity [60].

Although the robotic stimulus was substantially improved in its morphology and swimming pattern, it is unlikely that fish perceived it as a conspecific. Preference tests with conspecifics indicate a stronger attraction with a PI on the order of 70% [34], even if we should acknowledge that these studies permitted bidirectional interactions between the live stimulus and the focal subjects. Such interactions are not allowed in the present experimental set-up, where the replica is manoeuvred along pre-programmed trajectories, similar to preference tests in which live stimuli are separated using one-way glass [20]. Future work should seek to incorporate bidirectional interactions through a closed-loop control of the replica based on real-time data of fish motion using a similar approach to [36,38,47].

Other limiting factors of our robotic platform are the partial smoothness of the motion imparted to the rod, the mechanical rigidity of the replica and the rudimentary control of its orientation. Indirect evidence for the relevance of these locomotory aspects can be garnered by examining the increased activity of live fish in the presence of the moving replica, which may be associated with an anxiety-related response [14,52,66]. The smoothness of the trajectory could be improved by increasing the number of data points sampled from real fish trajectory. However, the density of such points should be sufficiently sparse to prevent jittering of the servomotors, owing to positioning uncertainties. The mechanical rigidity of the replica could be reduced by capitalizing on recent technological advances in three-dimensional printing [67], which have demonstrated the possibility of printing soft material. The use of a more compliant replica could help mimicking the body undulations of zebrafish, potentially enhancing the attraction towards the robotic stimulus. Enabling finer control of the orientation of the replica could be achieved by replacing the current servomotor on the travelling cantilever with a stepper motor (permitting unhindered continuous rotation and angle control), and synchronizing it with the rod three-dimensional motion, such that the replica orientation would align with its velocity vector.

Similar to video stimuli [22], a robotics-based approach enables the presentation of a consistent and customizable stimulus. However, in contrast with video stimuli, robots are three-dimensional physical objects and thus expected to better proxy live stimuli. This study offers further demonstration for the potential of robotics in the study of zebrafish social behaviour through a novel robotic platform that enables the presentation of a complex visual stimulus, consisting of a biologically inspired replica that is manoeuvred in three dimensions while oscillating its body.

## Supplementary Material

Supplementary material: Zebrafish response to a robotic replica in three dimensions

## Supplementary Material

Dataset 1

## Supplementary Material

Dataset 2

## Footnotes

1 If the same analysis were repeated using only the first 5 min of the experiment, then no significant information transfer would be detected, thereby suggesting the need for carefully selecting the duration of the observations for successful use of transfer entropy.

## Data accessibility

The original dataset supporting this article is available as electronic supplementary material.

## References

[1] Guo S 2004 Linking genes to brain, behavior and neurological diseases: what can we learn from zebrafish? Genes, Brain Behav. 3, 63–74. (doi:10.1046/j.1601-183X.2003.00053.x) 15005714 10.1046/j.1601-183x.2003.00053.x

[2] Golling G et al. 2002 Insertional mutagenesis in zebrafish rapidly identifies genes essential for early vertebrate development. Nat. Genet. 31, 135–140. (doi:10.1038/ng896) 12006978 10.1038/ng896

[3] Grunwald DJ, Eisen JS 2002 Headwaters of the zebrafish—emergence of a new model vertebrate. Nat. Rev. Genet. 3, 717–724. (doi:10.1038/nrg892) 12209146 10.1038/nrg892

[4] Nagel R 2001 *Dar*T: The embryo test with the zebrafish *Danio rerio*—a general model in ecotoxicology and toxicology. ALTEX 19, 38–48. 12096329

[5] Vittozzi L, De Angelis G 1991 A critical review of comparative acute toxicity data on freshwater fish. Aquat. Toxicol. 19, 167–204. (doi:10.1016/0166-445X(91)90017-4)

[6] Zon LI, Peterson RT 2005 *In vivo* drug discovery in the zebrafish. Nat. Rev. Drug Discov. 4, 35–44. (doi:10.1038/nrd1606) 15688071 10.1038/nrd1606

[7] Lieschke GJ, Currie PD 2007 Animal models of human disease: zebrafish swim into view. Nat. Rev. Genet. 8, 353–367. (doi:10.1038/nrg2091) 17440532 10.1038/nrg2091

[8] Green J et al. 2012 Automated high-throughput neurophenotyping of zebrafish social behavior. J. Neurosci. Methods 210, 266–271. (doi:10.1016/j.jneumeth.2012.07.017) 22884772 10.1016/j.jneumeth.2012.07.017

[9] Miklósi Á, Andrew RJ 2006 The zebrafish as a model for behavioral studies. Zebrafish 3, 227–234. (doi:10.1089/zeb.2006.3.227) 18248263 10.1089/zeb.2006.3.227

[10] Berger J, Currie P 2007 The role of zebrafish in chemical genetics. Curr. Med. Chem. 14, 2413–2420. (doi:10.2174/092986707781745532) 17896989 10.2174/092986707781745532

[11] Lawrence C 2007 The husbandry of zebrafish (*Danio rerio*): a review. Aquaculture 269, 1–20. (doi:10.1016/j.aquaculture.2007.04.077)

[12] Lam SH, Mathavan S, Tong Y, Li H, Karuturi RKM, Wu Y, Vega VB, Liu ET, Gong Z 2008 Zebrafish whole-adult-organism chemogenomics for large-scale predictive and discovery chemical biology. PLoS Genet. 4, e1000121 (doi:10.1371/journal.pgen.1000121) 18618001 10.1371/journal.pgen.1000121 PMC2442223

[13] Mathur P, Guo S 2010 Use of zebrafish as a model to understand mechanisms of addiction and complex neurobehavioral phenotypes. Neurobiol. Dis. 40, 66–72. (doi:10.1016/j.nbd.2010.05.016) 20493262 10.1016/j.nbd.2010.05.016 PMC3021971

[14] Kalueff AV et al. 2013 Towards a comprehensive catalog of zebrafish behavior 1.0 and beyond. Zebrafish 10, 70–86. (doi:10.1089/zeb.2012.0861) 23590400 10.1089/zeb.2012.0861 PMC3629777

[15] Stewart AM et al. 2010 The developing utility of zebrafish in modeling neurobehavioral disorders. Int. J. Comp. Psychol. 23, 104–120. (doi:10.1016/j.pnpbp.2013.11.014)

[16] Gerlai R 2012 Using zebrafish to unravel the genetics of complex brain disorders. In Behavioral neurogenetics (eds Cryan JF, Reif A), pp. 3–24. Berlin, Germany: Springer. 10.1007/7854_2011_180 22250005

[17] Kalueff AV, Stewart AM, Gerlai R 2014 Zebrafish as an emerging model for studying complex brain disorders. Trends Pharmacol. Sci. 35, 63–75. (doi:10.1016/j.tips.2013.12.002) 24412421 10.1016/j.tips.2013.12.002 PMC3913794

[18] Stewart AM, Braubach O, Spitsbergen J, Gerlai R, Kalueff A 2014 Zebrafish models for translational neuroscience research: from tank to bedside. Trends Neurosci. 37, 264–278. (doi:10.1016/j.tins.2014.02.011) 24726051 10.1016/j.tins.2014.02.011 PMC4039217

[19] Krause J, Butlin RK, Peuhkuri N, Pritchard VL 2000 The social organization of fish shoals: a test of the predictive power of laboratory experiments for the field. Biol. Rev. 75, 477–501. (doi:10.1111/j.1469-185X.2000.tb00052.x) 11117198 10.1111/j.1469-185x.2000.tb00052.x

[20] Wright D, Krause J 2006 Repeated measures of shoaling tendency in zebrafish (*Danio rerio*) and other small teleost fishes. Nat. Protoc. 1, 1828–1831. (doi:10.1038/nprot.2006.287) 17487165 10.1038/nprot.2006.287

[21] Woo KL, Rieucau G 2011 From dummies to animations: a review of computer-animated stimuli used in animal behavior studies. Behav. Ecol. Sociobiol. 65, 1671–1685. (doi:10.1007/s00265-011-1226-y)

[22] Qin M, Wong A, Seguin D, Gerlai R 2014 Induction of social behavior in zebrafish: live versus computer animated fish as stimuli. Zebrafish 11, 185–197. (doi:10.1089/zeb.2013.0969) 24575942 10.1089/zeb.2013.0969 PMC4050712

[23] Gerlai R 2010 High-throughput behavioral screens: the first step towards finding genes involved in vertebrate brain function using zebrafish. Molecules 15, 2609–2622. (doi:10.3390/molecules15042609) 20428068 10.3390/molecules15042609 PMC6257226

[24] Engeszer RE, Ryan MJ, Parichy DM 2004 Learned social preference in zebrafish. Curr. Biol. 14, 881–884. (doi:10.1016/j.cub.2004.04.042) 15186744 10.1016/j.cub.2004.04.042

[25] Saverino C, Gerlai R 2008 The social zebrafish: behavioral responses to conspecific, heterospecific, and computer animated fish. Behav. Brain Res. 191, 77–87. (doi:10.1016/j.bbr.2008.03.013) 18423643 10.1016/j.bbr.2008.03.013 PMC2486438

[26] Pham M et al. 2012 Assessing social behavior phenotypes in adult zebrafish: shoaling, social preference, and mirror biting tests. In Zebrafish protocols for neurobehavioral research (eds Kalueff A, Stewart AM), pp. 231–246. Totowa, NJ: Humana Press.

[27] Polverino G, Porfiri M 2013 Zebrafish (*Danio rerio*) behavioural response to bioinspired robotic fish and mosquitofish (*Gambusia affinis*). Bioinspir. Biomim. 8, 044001 (doi:10.1088/1748-3182/8/4/044001) 23999758 10.1088/1748-3182/8/4/044001

[28] Ruhl N, McRobert SP 2005 The effect of sex and shoal size on shoaling behaviour in *Danio rerio*. J. Fish Biol. 67, 1318–1326. (doi:10.1111/j.0022-1112.2005.00826.x)

[29] Turnell ER, Mann KD, Rosenthal GG, Gerlach G 2003 Mate choice in zebrafish (*Danio rerio*) analyzed with video-stimulus techniques. Biol. Bull. 205, 225–226. (doi:10.2307/1543265) 14583542 10.2307/1543265

[30] Abaid N, Spinello C, Laut J, Porfiri M 2012 Zebrafish (*Danio rerio*) responds to images animated by mathematical models of animal grouping. Behav. Brain Res. 232, 406–410. (doi:10.1016/j.bbr.2012.03.028) 22469628 10.1016/j.bbr.2012.03.028

[31] Krause J, Winfield AF, Deneubourg JL 2011 Interactive robots in experimental biology. Trends Ecol. Evol. 26, 369–375. (doi:10.1016/j.tree.2011.03.015) 21496942 10.1016/j.tree.2011.03.015

[32] Butail S, Abaid N, Macrì S, Porfiri M 2015 Fish–robot interactions: robot fish in animal behavioral studies. In Robot Fish, pp. 221–240, part IV Berlin, Germany: Springer.

[33] Ladu F, Bartolini T, Panitz SG, Chiarotti F, Butail S, Macri S, Porfiri M 2015 Live predators, robots, and computer-animated images elicit differential avoidance responses in zebrafish. Zebrafish 12, 205–214. (doi:10.1089/zeb.2014.1041) 25734228 10.1089/zeb.2014.1041

[34] Spinello C, Macrì S, Porfiri M 2013 Acute ethanol administration affects zebrafish preference for a biologically inspired robot. Alcohol 47, 391–398. (doi:10.1016/j.alcohol.2013.04.003) 23725654 10.1016/j.alcohol.2013.04.003

[35] Landgraf T, Nguyen H, Schröer J, Szengel A, Clément RJG, Bierbach D, Krause J 2014 Blending in with the shoal: robotic fish swarms for investigating strategies of group formation in guppies. In Biomimetic and biohybrid systems, pp. 178–189. Berlin, Germany: Springer.

[36] Landgraf T, Bierbach D, Nguyen H, Muggelberg N, Romanczuk P, Krause J 2016 RoboFish: increased acceptance of interactive robotic fish with realistic eyes and natural motion patterns by live Trinidadian guppies. Bioinspir. Biomim. 11, 015001 (doi:10.1088/1748-3190/11/1/015001) 26757096 10.1088/1748-3190/11/1/015001

[37] Marras S, Killen SS, Lindström J, McKenzie DJ, Steffensen JF, Domenici P 2015 Fish swimming in schools save energy regardless of their spatial position. Behav. Ecol. Sociobiol. 69, 219–226. (doi:10.1007/s00265-014-1834-4) 25620833 10.1007/s00265-014-1834-4 PMC4293471

[38] Swain DT, Couzin ID, Leonard NE 2012 Real-time feedback-controlled robotic fish for behavioral experiments with fish schools. Proc. IEEE 100, 150–163. (doi:10.1109/JPROC.2011.2165449)

[39] Polverino G, Phamduy P, Porfiri M 2013 Fish and robots swimming together in a water tunnel: robot color and tail-beat frequency influence fish behavior. PLoS ONE 8, e77589 (doi:10.1371/journal.pone.0077589) 24204882 10.1371/journal.pone.0077589 PMC3808421

[40] Polverino G, Porfiri M 2013 Mosquitofish (*Gambusia affinis*) responds differentially to a robotic fish of varying swimming depth and aspect ratio. Behav. Brain Res. 250, 133–138. (doi:10.1016/j.bbr.2013.05.008) 23684918 10.1016/j.bbr.2013.05.008

[41] Faria JJ, Dyer JRG, Clément RO, Couzin ID, Holt N, Ward AJW, Waters D, Krause J 2010 A novel method for investigating the collective behaviour of fish: introducing ‘Robofish’. Behav. Ecol. Sociobiol. 64, 1211–1218. (doi:10.1007/s00265-010-0988-y)

[42] Phamduy P, Polverino G, Fuller RC, Porfiri M 2014 Fish and robot dancing together: bluefin killifish females respond differently to the courtship of a robot with varying color morphs. Bioinspir. Biomim. 9, 036021 (doi:10.1088/1748-3182/9/3/036021) 25162832 10.1088/1748-3182/9/3/036021

[43] Aureli M, Fiorilli F, Porfiri M 2012 Portraits of self-organization in fish schools interacting with robots. Physica D: Nonlinear Phenomena 241, 908–920. (doi:10.1016/j.physd.2012.02.005)

[44] Abaid N, Bartolini T, Macri S, Porfiri M 2012 Zebrafish responds differentially to a robotic fish of varying aspect ratio, tail beat frequency, noise, and color. Behav. Brain Res. 233, 545–553. (doi:10.1016/j.bbr.2012.05.047) 22677270 10.1016/j.bbr.2012.05.047

[45] Polverino G, Abaid N, Kopman V, Macrì S, Porfiri M 2012 Zebrafish response to robotic fish: preference experiments on isolated individuals and small shoals. Bioinspir. Biomim. 7, 036019 (doi:10.1088/1748-3182/7/3/036019) 22677608 10.1088/1748-3182/7/3/036019

[46] Cianca V, Bartolini T, Porfiri M, Macri S 2013 A robotics-based behavioral paradigm to measure anxiety-related responses in zebrafish. PLoS ONE 8, e69661 (doi:10.1371/journal.pone.0069661) 23922773 10.1371/journal.pone.0069661 PMC3726767

[47] Kopman V, Laut J, Polverino G, Porfiri M 2013 Closed-loop control of zebrafish response using a bioinspired robotic-fish in a preference test. J. R. Soc. Interface 10, 20120540 (doi:10.1098/rsif.2012.0540) 23152102 10.1098/rsif.2012.0540 PMC3565779

[48] Bartolini T, Mwaffo V, Showler A, Macrì S, Butail S, Porfiri M 2016 Zebrafish response to 3D printed shoals of conspecifics: the effect of body size. Bioinspir. Biomim. 11, 026003 (doi:10.1088/1748-3190/11/2/026003) 26891476 10.1088/1748-3190/11/2/026003

[49] Butail S, Ladu F, Spinello D, Porfiri M 2014 Information flow in animal–robot interactions. Entropy 16, 1315–1330. (doi:10.3390/e16031315)

[50] Cachat J, Stewart A, Utterback E, Hart P, Gaikwad S, Wong K, Kyzar E, Wu N, Kalueff AV 2011 Three-dimensional neurophenotyping of adult zebrafish behavior. PLoS ONE 6, e17597 (doi:10.1371/journal.pone.0017597) 21408171 10.1371/journal.pone.0017597 PMC3049776

[51] Mikut R et al. 2013 Automated processing of zebrafish imaging data: a survey. Zebrafish 10, 401–421. (doi:10.1089/zeb.2013.0886) 23758125 10.1089/zeb.2013.0886 PMC3760023

[52] Stewart AM, Gaikwad S, Kyzar E, Green J, Roth A, Kalueff AV 2012 Modeling anxiety using adult zebrafish: a conceptual review. Neuropharmacology 62, 135–143. (doi:10.1016/j.neuropharm.2011.07.037) 21843537 10.1016/j.neuropharm.2011.07.037 PMC3195883

[53] Schreiber T 2000 Measuring information transfer. Phys. Rev. Lett. 85, 461 (doi:10.1103/PhysRevLett.85.461) 10991308 10.1103/PhysRevLett.85.461

[54] Ladu F, Mwaffo V, Li J, Macri S, Porfiri M 2015 Acute caffeine administration affects zebrafish response to a robotic stimulus. Behav. Brain Res. 289, 48–54. (doi:10.1016/j.bbr.2015.04.020) 25907748 10.1016/j.bbr.2015.04.020

[55] Butail S, Bartolini T, Porfiri M 2013 Collective response of zebrafish shoals to a free-swimming robotic fish. PLoS ONE 8, e76123 (doi:10.1371/journal.pone.0076123) 24146825 10.1371/journal.pone.0076123 PMC3797741

[56] Cahill GM 1996 Circadian regulation of melatonin production in cultured zebrafish pineal and retina. Brain Res. 708, 177–181. (doi:10.1016/0006-8993(95)01365-2) 8720875 10.1016/0006-8993(95)01365-2

[57] Ward AJW, Thomas P, Hart PJB, Krause J 2004 Correlates of boldness in three-spined sticklebacks (*Gasterosteus aculeatus*). Behav. Ecol. Sociobiol. 55, 561–568. (doi:10.1007/s00265-003-0751-8)

[58] Ruddell BL, Kumar P 2009 Ecohydrologic Process Networks: 1. Identification. Water Resour. Res. 45, W03419 (doi:10.1029/2008WR007279)

[59] Miller JN 1993 Tutorial review—outliers in experimental data and their treatment. Analyst 118, 455–461. (doi:10.1039/AN9931800455)

[60] Pita D, Moore BA, Tyrrell LP, Fernández-Juricic E 2015 Vision in two cyprinid fish: implications for collective behavior. PeerJ 3, e1113 (doi:10.7717/peerj.1113) 26290783 10.7717/peerj.1113 PMC4540049

[61] Hu F, Nie LJ, Fu SJ 2015 Information dynamics in the interaction between a prey and a predator fish. Entropy 17, 7230–7241. (doi:10.3390/e17107230)

[62] Orange N, Abaid N 2015 A transfer entropy analysis of leader-follower interactions in flying bats. Eur. Phys. J. Spec. Top. 224, 3279–3293. (doi:10.1140/epjst/e2015-50235-9)

[63] Butail S, Mwaffo V, Porfiri M 2016 Model-free information-theoretic approach to infer leadership in pairs of zebrafish. Phys. Rev. E 93, 042411 (doi:10.1103/PhysRevE.93.042411) 27176333 10.1103/PhysRevE.93.042411

[64] Wang XR, Miller JM, Lizier JT, Prokopenko M, Rossi LF 2012 Quantifying and tracing information cascades in swarms. PLoS ONE 7, e40084 (doi:10.1371/journal.pone.0040084) 22808095 10.1371/journal.pone.0040084 PMC3395630

[65] Sun Y, Rossi LF, Shen CC, Miller JN, Wang XR, Lizier JT, Prokopenko M, Senanayake U 2014 Information transfer in swarms with leaders. (arXiv:1407.0007)

[66] Maximino C, de Brito TM, da Silva Batista AW, Herculano AM, Morato S, Gouveia A 2010 Measuring anxiety in zebrafish: a critical review. Behav. Brain Res. 214, 157–171. (doi:10.1016/j.bbr.2010.05.031) 20510300 10.1016/j.bbr.2010.05.031

[67] Lipson H, Kurman M 2013 Fabricated: the new *world of 3D printing* Indianapolis, IN: John Wiley & Sons.

