# Supplementary material for: Zebrafish response to a robotic replica in three dimensions
Source: R Soc Open Sci. 2016 Oct 19;3(10):160505. doi: 10.1098/rsos.160505 (PMC5098991; doi:10.1098/rsos.160505)
Supplement: Supplementary material: Zebrafish response to a robotic replica in three dimensions [file rsos160505supp1.docx]

**Supplementary material: Zebrafish response to a robotic replica in three dimensions**

Tommaso Ruberto^1^, Violet Mwaffo^1^, Sukhgewanpreet Singh^1^, Daniele Neri^1^, Maurizio Porfiri^1*^

*^1^Department of Mechanical and Aerospace Engineering, New York University Tandon School of Engineering, Brooklyn, New York, 11201, USA*

^*^Corresponding author: [mporfiri@nyu.edu](mailto:mporfiri@nyu.edu), 646-997-3681 (phone), 646-997-3532 (fax)

1. **Top/front sample video of one experiment of RM condition**

The sample video shows 60 s of one trial for “replica + motion”, RM, condition.

1. **Original supporting dataset 1 and 2**

The original dataset supporting this article consists of two excel files, including all the values for the indicators analysed in the article.
